# Long-Lasting Cross-Protection Against Influenza A by Neuraminidase and M2e-based immunization strategies

**DOI:** 10.1038/srep24402

**Published:** 2016-04-13

**Authors:** Michael Schotsaert, Tine Ysenbaert, Anouk Smet, Bert Schepens, Dieter Vanderschaeghe, Svetlana Stegalkina, Thorsten U. Vogel, Nico Callewaert, Walter Fiers, Xavier Saelens

**Affiliations:** 1 Medical Biotechnology Center, VIB, Ghent, 9052, Belgium; 2 Department of Biomedical Molecular Biology, Ghent University, Ghent, 9052, Belgium; 3 Department of Biochemistry and Microbiology, Ghent University, Ghent, 9052, Belgium; 4 Sanofi Pasteur, Research North America, Cambridge, Massachusetts, USA

## Abstract

There is mounting evidence that in the absence of neutralizing antibodies cross-reactive T cells provide protection against pandemic influenza viruses. Here, we compared protection and CD8+ T cell responses following challenge with H1N1 2009 pandemic and H3N2 viruses of mice that had been immunized with hemagglutinin (HA), neuraminidase (NA) and the extracellular domain of matrix protein 2 (M2e) fused to a virus-like particle (VLP). Mice were challenged a first time with a sublethal dose of H1N1 2009 pandemic virus and, four weeks later, challenged again with an H3N2 virus. Mice that had been vaccinated with HA, NA, NA + M2e-VLP and HA + NA + M2e-VLP were protected against homologous H1N1 virus challenge. Challenged NA and NA + M2e-VLP vaccinated mice mounted CD8+ T cell responses that correlated with protection against secondary H3N2 challenge. HA-vaccinated mice were fully protected against challenge with homologous H1N1 2009 virus, failed to mount cross-reactive CD8+ T cells and succumbed to the second challenge with heterologous H3N2 virus. In summary, NA- and M2e-based immunity can protect against challenge with (homologous) virus without compromising the induction of robust cross-reactive CD8+ T cell responses upon exposure to virus.

Human influenza remains a major health problem affecting all age groups worldwide. Current influenza vaccines aim at neutralizing virus by the induction of antibodies that target the globular head of the hemagglutinin (HA) protein, the receptor-binding protein. Neutralization of influenza viruses by antibodies that target HA is an effective strategy when HA in the vaccine antigenically matches the HA of circulating viruses. This requires regular vaccine updates as human influenza HA is prone to genetic drift. Influenza infection of an immunologically naïve host triggers robust CD8+ T cell responses that are broadly reactive. Those CD8+ T cells are essential to clear influenza viruses from the lungs and, given their broad antigen-specificity, can reduce disease caused by a pandemic influenza A virus outbreak^1^,^2^,^3^. Indeed, correlations between productive virus infection and cross-reactive cellular immunity against subsequent infection with heterologous influenza viruses have been reported based on studies involving humans, monkeys, ferrets and mice^2^,^3^,^4^,^5^,^6^,^7^,^8^. In addition, cytotoxic T cells can protect against influenza A virus and correlate with faster virus clearance and reduced virus shedding upon experimental challenge^2^,^6^,^9^,^10^. Preventing virus replication by neutralizing antibodies may hinder *de novo* viral gene expression and presentation of these gene products to B and T cells. Observational and experimental studies in different animal models suggest that immunization with inactivated influenza vaccines interferes with induction of cross-reactive CD8+ T cells in response to infection^8^,^11^,^12^,^13^.

Yearly vaccination of infants during their first year of life with licensed influenza vaccines is now recommended in different countries (http://www.cdc.gov/flu/protect/children.htm, http://www.phac-aspc.gc.ca/naci-ccni/flu-grippe-eng.php). This target group can be considered immunologically naïve for influenza. Assuming a vaccine effectiveness of over 50%, virus replication and disease would be significantly reduced in this age group and likely concomitantly hamper the induction of influenza-specific CD8+ T cells early in life^14^.

Influenza A viruses encode three membrane proteins: HA, NA and matrix protein 2 (M2). Inspired by the identification of monoclonal antibodies that target conserved epitopes in the stalk of HA^15^,^16^, many research groups are focusing on novel HA-based vaccination strategies to induce or boost this type of immunity^17^,^18^,^19^. These type of monoclonal antibodies can neutralize influenza viruses *in vitro* but rely on Fcγ Receptors to protect *in vivo*^20^. Antibodies against the surface exposed part of NA and M2 (referred to as M2e) also protect in experimental influenza A virus challenge models^21^,^22^,^23^. Thanks to its strong sequence conservation M2e is a candidate universal influenza A vaccine antigen that has proven safe and immunogenic in a phase I clinical trial^24^. In animal models, these types of broadly protective antibodies prevent severe disease but do not provide sterilizing immunity. We recently showed that immunization with an M2e-based vaccine provided protection upon primary challenge but allowed potent priming of cross-reactive T cells, which strengthened protection against secondary infection with a heterosubtypic virus^13^. We and others also showed that sterilizing immunity provided by vaccination with a whole inactivated virus vaccine was associated with strongly reduced induction of these protective cross-reactive T cell responses upon infection with an antigenically matching virus^13^,^25^.

Immunity to NA, the second viral glycoprotein, is considered infection-permissive^21^ and correlates with protection^21^,^23^,^26^,^27^,^28^,^29^,^30^. NA is a mushroom-like, tetrameric type II membrane protein that is necessary to release new progeny virus from the infected cell by removing terminal sialic acids from budding virions and the cell surface^31^,^32^. NA of human influenza viruses is subject to antigenic drift and antigenic shift suggesting that it is under strong immune selection pressure in humans^33^,^34^,^35^. Serum antibodies that inhibit NA activity correlated with protection against infection-associated illness in the first two years of the 2009 H1N1 pandemic^36^ and NA inhibitory antibodies were a much better correlate of protection against disease in a ferret model than anti-HA antibodies were, even though the latter correlated with reduced viral loads in the lungs^37^.

Here, we evaluated protection by three recombinant influenza A vaccine antigens: soluble tetrameric NA (tetNA), soluble trimeric HA (triHA), both derived from 2009 H1N1 pandemic virus, and M2e-displaying virus-like particles (M2e-VLP). We evaluated protection induced by immunization with tetNA, triHA and M2e-VLP, separately and combined, against challenge with homologous and heterosubtypic influenza virus in mouse. We found that the infection-permissive immunity provided by tetNA- and M2e-based vaccines protected against body weight loss and significantly reduced lung virus loads after challenge with 2009 H1N1 pandemic virus (A/Belgium/2009, hereafter designated as H1N1v). The universal M2e-based vaccine, alone or combined with tetNA, also protected against a challenge with H3N2 virus. Primary challenge of animals that had been immunized with tetNA, M2e-VLP or a combination of both was associated with the induction of robust CD8+ T cell responses directed against the viral nucleoprotein (NP). These cross-reactive T cell responses correlated with protection against subsequent challenge with an H3N2 virus.

## Results

### Expression, purification and characterization of soluble trimeric HA and tetrameric NA

**Figure 1:**
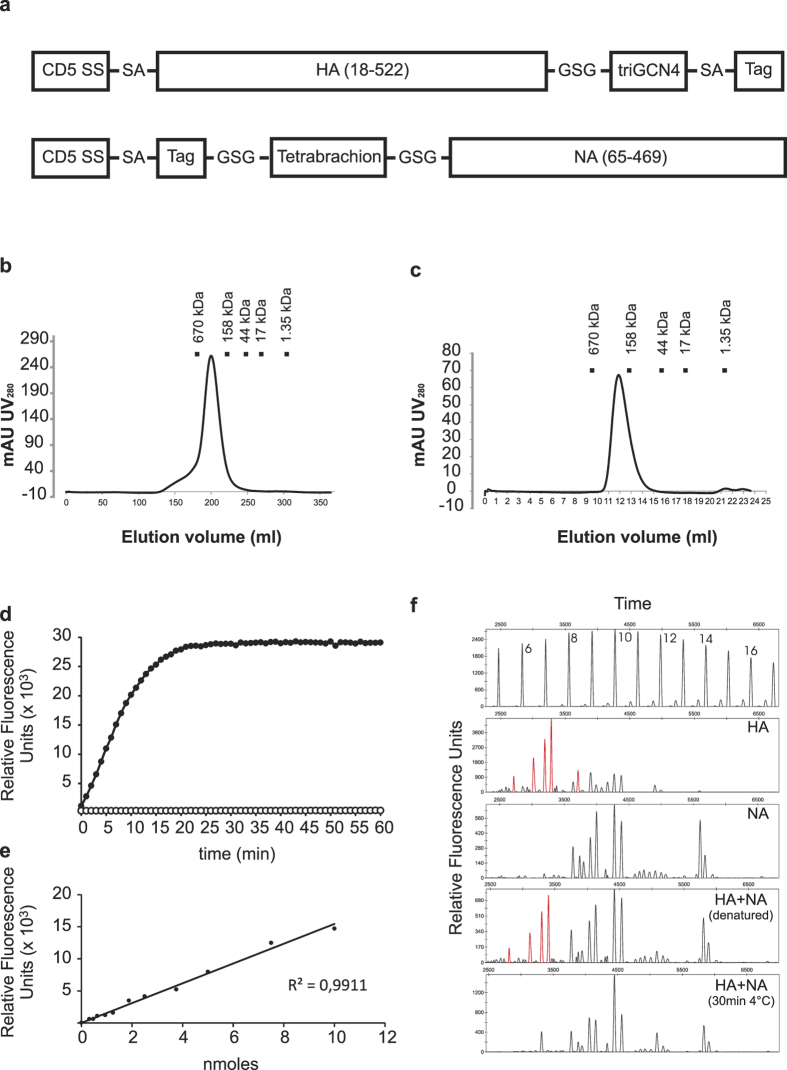
Design and characterization of recombinant soluble trimeric hemagglutinin (triHA) and tetrameric neuraminidase (tetNA) antigens used for vaccination. (**a**) Schematic representation of triHA and tetNA expression constructs. Amino acid residues separating the building blocks of the constructs are shown in single letter code. CD5-SS: secretion signal of CD5; triGCN4: trimerization leucine zipper derived from *Saccharomyces cerevisae* GCN4; Tag: Strep-Tag; tetrabrachion: tetramerization coiled coil derived from *Staphylothermus marinus* tetrabrachion. (**b,c**) UV absorption profile after size exclusion chromatography of triHA (**b**) and tetNA (**c**). (**d**) Enzymatic activity of tetNA in culture medium from pEF-NA transfected HEK293T cells (closed symbols) or culture medium only (open symbols) as measured by kinetic release of fluorescent 4-Methylumbelliferone from the 2′-(4-Methylumbelliferyl)-α-D-N-acetylneuraminic substrate. (**e**) Fluorescence calibration curve for calculation of released 4-Methylumbelliferone. (**f**) Neuraminidase activity of tetNA results in desialylation of triHA N-glycans when mixed under conditions of vaccine formulation. Top panel shows a dextran ladder as a reference for the glycan structures, numbers represent glucose units. Other panels show N-linked glycan profiles for triHA (HA), tetNA (NA), triHA + tetNA in the presence of 8 M Urea (HA + NA (denatured)) and triHA + tetNA under vaccine formulation conditions (30 min at 4 °C) (HA + NA). Peaks in red indicate glycans on triHA that are desialylated in the presence of native tetNA.

We produced and purified recombinant, soluble oligomeric HA (triHA) and NA (tetNA) derived from H1N1v. To ensure correct oligomerisation and stability in the absence of the transmembrane domains, we genetically fused the extracellular domain of HA (HA 18–522) at the C-terminus to a trimerization GCN4-derived leucine zipper, and the extracellular domain of NA (NA 65–469), without the stalk, at the N-terminus to the tetramerization tetrabrachion coiled coil (Fig. 1a). TriHA and tetNA were produced in HEK293T cells and affinity purified from the cleared culture supernatant followed by a polishing step by size exclusion chromatography. The predominant peak of triHA eluted with a retention volume corresponding to glycosylated trimeric HA (Fig. 1b). A minor fraction, which was discarded, eluted faster and likely corresponded to higher order complexes of triHA (Fig. 1b). Gel filtration analysis of tetNA revealed a single peak that corresponded to the predicted molecular weight of soluble tetrameric NA (Fig. 1c). TetNA was enzymatically active and had a specific activity of 11,000 pmole/min/μg as determined by a fluorimetric assay using 2′-4-Methylumbelliferyl-α-D-N-acetylneuraminic acid as substrate (Fig. 1d,e). We also analyzed the N-glycan composition of purified triHA and tetNA using DNA-sequencer-assisted fluorophore-assisted carbohydrate electrophoresis^38^. Recombinant triHA contained sialylated N-glycans (Fig. 1f). In contrast, purified tetNA only contained neutral, non-sialylated N-glycans (Fig. 1f), most probably because of self desialylation. We also analyzed the N-glycan profiles of triHA and tetNA mixed together in equal amounts (mass) in phosphate buffered saline and incubated at 4 °C for 30 min. Even though incubation was at 4 °C, HA was almost completely desialylated by tetNA under these conditions (Fig. 1f). Prior denaturation of tetNA with 8 M urea did not affect the sialylation status of co-incubated triHA, indicating that enzymatically active tetNA was responsible for the desialylation of triHA. In summary, we obtained pure, soluble HA and NA with a natural oligomeric conformation. Furthermore, tetNA was enzymatically active.

### NA-based immunity provided by vaccination is infection-permissive

**Figure 2:**
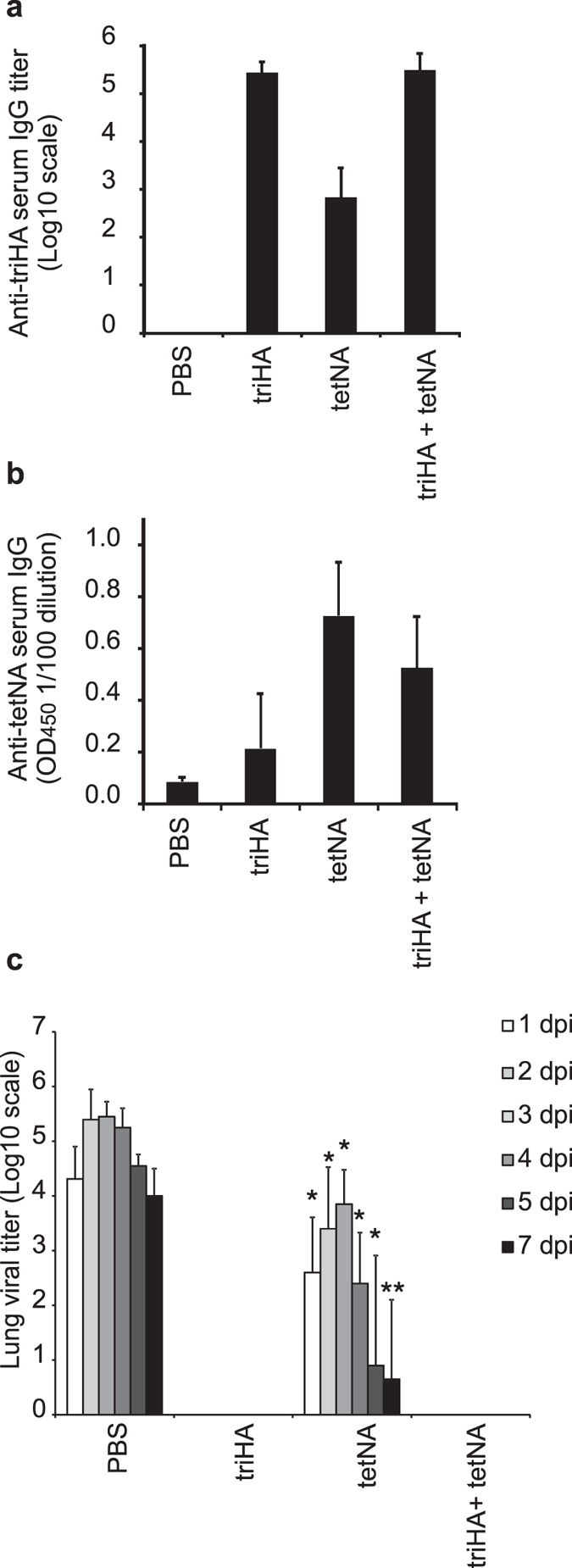
NA-specific immunity permits reduced virus replication. Female 6–8 weeks old BALB/c mice (n = 30/group) were immunized twice with 1μg triHA, tetNA or triHA + tetNA formulated with SAS adjuvant. Seroconversion was determined with serum collected two weeks after the booster vaccination by ELISA and (**a**) expressed as endpoint serum total IgG ELISA titers directed against coated triHA or (**b**) as OD_450_ of a 1/100 serum dilution directed against coated tetNA. (**c**) After intranasal challenge with 0.1LD_50_ H1N1v, lung virus titers were determined by endpoint titration on MDCK cells in cleared lung homogenates (n = 5/day/group) harvested at different days post infection (dpi). Lung virus loads are expressed as mean ± SD of tissue culture infectious dose 50 (TCID50) per gram of lung extract. Lung virus titers for triHA and triHA + tetNA were below detection limit. Bars represent averages and error bars standard deviations. *P < 0.05 and **P < 0.01 compared to PBS (two-sided Mann-Whitney U test with correction for ties).

The canonical view is that IgG targeting influenza NA primarily interferes with virion release from infected cells and therefore NA-based immunity is considered infection-permissive^39^,^40^,^41^. To test the infection-permissive nature of NA-specific immunity, we performed a pilot experiment in which lung virus loads in mice that had been vaccinated with triHA, tetNA or a combination of both at different time points after infection with 0.1 LD_50_ of homologous mouse-adapted H1N1v virus. The mice (n = 30 for each group) seroconverted to the vaccine antigens (Fig. 2a,b). triHA and tetNA share the Streptag which likely explains why vaccination with triHA and tetNA resulted in low serum IgG in tetNA mice against triHA (Fig. 2a) and reciprocally low serum reactivity of triHA immunized mice against tetNA (Fig. 2b). When mice were challenged three weeks after the booster immunization with H1N1v, no detectable virus titers were observed at any time point after H1N1v challenge in mice that had been vaccinated with triHA or triHA + tetNA (Fig. 2c). NA-specific immunity on the other hand was infection-permissive as mice vaccinated with tetNA had detectable virus titers until 7 days post infection. However, lung virus titers were significantly lower in NA-immune mice compared to PBS + adjuvant immunized mice (Fig. 2c).

### Vaccination with triHA, tetNA, M2e-VLP and combinations thereof protects against primary homologous challenge

**Figure 3:**
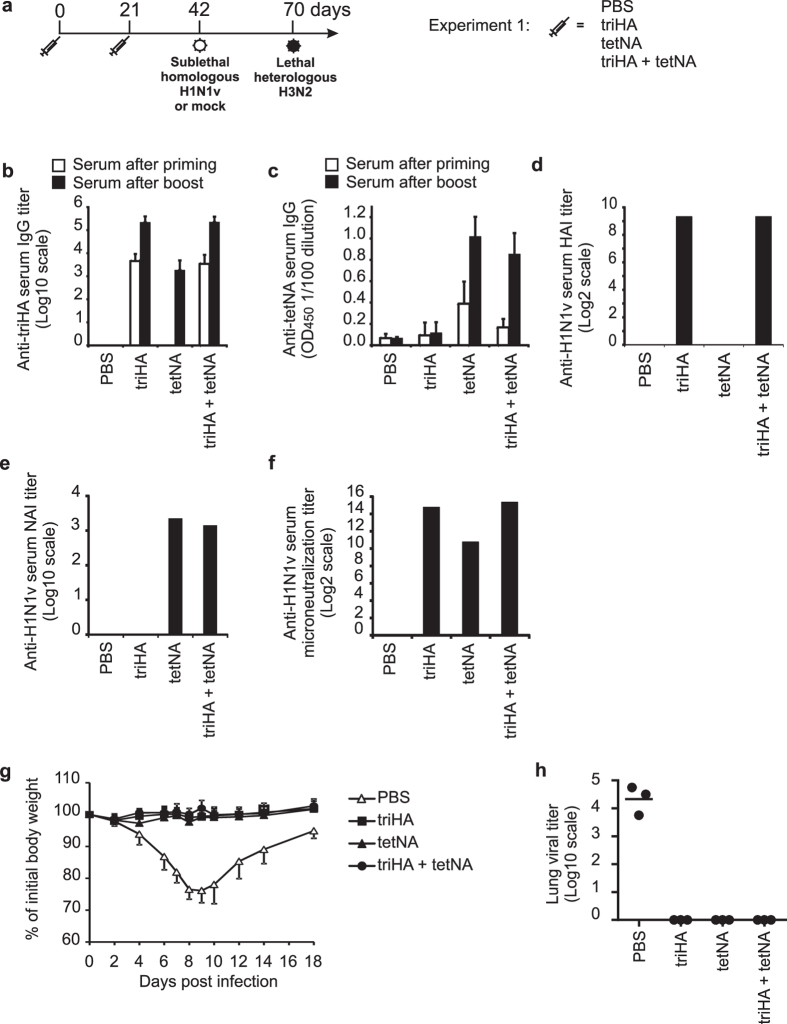
(**a**) Outline of Experiment 1. Groups of 62 female SPF BALB/c mice (6–8 weeks old) were immunized two times subcutaneously with the indicated antigens in the presence of SAS. Three weeks after the booster immunization, half of the mice in each group were challenged with a sublethal dose of H1N1v (0.1 LD_50_) and the other mice were mock-challenged. Four weeks after the primary challenge, all mice were rechallenged with a lethal dose (2 LD_50_) of mouse-adapted X47 (H3N2) virus. Following challenge, body weight and survival were monitored and some of the mice were sacrificed for lung and spleen sampling. Seroconversion after prime/boost vaccination of female BALB/c mice (6–8 weeks old) with 1μg triHA, 1μg tetNA or 1μg triHA + 1μg tetNA formulated with SAS adjuvant (from experiment 1, n = 62 mice/group) was determined by ELISA and (**b**) expressed as endpoint serum total IgG ELISA titers for triHA or (**c**) as OD_450_ of a 1/100 serum dilution for tetNA. In (**b**,**c**) bars represent averages and error bars standard deviations. (**d**) Serum HAI titer against homologous pandemic H1N1v virus in pooled sera (n = 10) for each group collected two weeks after booster vaccination. (**e**) NAI titer determined with tetNA as source of sialidase in pooled sera (n = 10) for each group collected two weeks after booster vaccination. (**f**) Microneutralization titer against homologous pandemic H1N1v virus determined with pooled sera (n = 10) for each group collected two weeks after booster vaccination. (**g**) Protection by vaccination with triHA, tetNA or triHA + tetNA against homologous challenge with 0.1LD_50_ of H1N1v virus (n = 12 mice/group). PBS-vaccinated mice showed significantly more body weight loss between 4 and 18 dpi compared to triHA-, tetNA- or triHA + tetNA mice (two-sided Mann-Whitney *U* test with correction for ties for each dpi. P value < 0.01). (**h**) Lung virus titers (n = 3/group) harvested at 6 dpi. Lung virus loads are expressed as tissue culture infectious dose 50 (TCID_50_) per gram of lung extract. Individual virus titers are given and lines represent averages. Lung virus titers in triHA, tetNA and triHA + tetNA were below the detection limit.

A first objective of this study was to evaluate if immunization with different recombinant antigens, all derived from the extracellular domains of the viral membrane proteins, could protect mice against primary homologous (with respect to HA and NA antigens, both H1N1v-derived) and against primary heterosubtypic (H3N2) influenza A virus challenge. A second objective was to evaluate how the immune response upon the primary challenge of control and vaccinated mice affects the outcome of a second challenge with the heterosubtypic H3N2 virus (Fig. 3a). The first challenge, three weeks after the boost vaccination, was with 0.1 LD_50_ of H1N1v. In addition, half of the mice in each group were mock-infected at this time point. The H3N2 challenge was given at day 70 (i.e. 4 weeks after the mock or H1N1v challenge) at a dose of 2LD_50_.

We initially tested the protective capacity of vaccination with tetNA, triHA or a combination of both antigens against challenge with the two virus subtypes. Groups of 62 BALB/c mice were immunized twice with three weeks interval. Vaccination resulted in seroconversion against the respective antigens as determined by ELISA, HAI, NAI and microneutralisation assays (Fig. 3b–f). The microneutralization activity of tetNA-immune is in line with a recent report in which an raccoon poxvirus-vectored H5N1-derived NA vaccine was evaluated (Fig. 3f)^42^. Next, half (31 out of 62) of the mice from each group were challenged with a sublethal dose of H1N1v and the other half of the mice were mock-infected. None of the triHA, tetNA and triHA plus tetNA immunized mice displayed body weight loss, whereas adjuvant only immunized mice suffered significant body weight loss until day 9 after challenge and then recovered (Fig. 3g). Furthermore, at day 6 after challenge PBS-vaccinated control animals had significant lung virus loads, whereas no residual virus was detectable in triHA and tetNA immunized mice (Fig. 3h).

**Figure 4:**
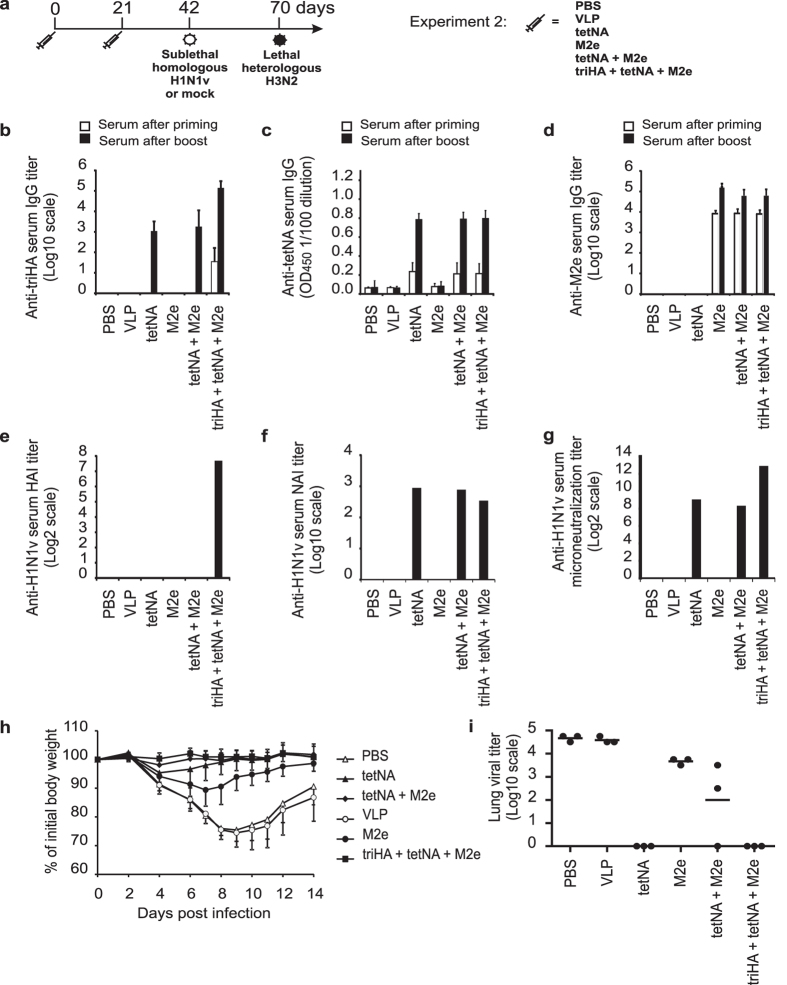
(**a**) Outline of Experiment 2. Groups of 62 female SPF BALB/c mice (6–8 weeks old) were immunized two times subcutaneously with the indicated antigens in the presence of SAS. Mice were challenged, monitored and sampled as described in Fig. 3. Seroconversion after prime/boost vaccination (n = 62/group) was determined by ELISA using (**b**) coated triHA and expressed as endpoint serum total IgG ELISA titers for triHA, (**c**) as OD_450_ of a 1/100 serum dilution against coated tetNA or (**d**) as endpoint serum total IgG ELISA titers using coated M2e peptide. (**e**) HAI titer against homologous pandemic H1N1v virus. (**f**) NAI titer determined with tetNA as source of sialidase. (**g**) Microneutralization titer against homologous pandemic H1N1v virus. In (**b**), (**c**) and (**d**) bars represent averages and error bars represent standard deviations. In (**e**,**f**,**g**) bars are from pooled sera (n = 10/group) isolated two weeks after booster vaccination. “M2e” in the X-axis means M2e-VLP. (**h**) Protection by vaccination with tetNA, M2e-VLP, tetNA + M2e-VLP or triHA + tetNA + M2e-VLP against homologous challenge with 0.1LD_50_ of H1N1v virus three weeks after booster vaccination (n = 12 mice/group). PBS-vaccinated mice (control for tetNA-vaccination) showed significantly more body weight loss between 2 and 14 dpi compared to tetNA-, tetNA + M2e-VLP or triHA + tetNA + M2e-VLP mice (two-sided Mann-Whitney *U* test with correction for ties for each dpi. P value < 0.05). Mice vaccinated with VLP (control for M2e-VLP-vaccination) showed significantly more body weight loss between 4 and 14 days after infection compared to M2e-VLP-, tetNA + M2e-VLP or triHA + tetNA + M2e-VLP mice (two-sided Mann-Whitney *U* test with correction for ties for each dpi. P value < 0.01). (**i**) Lung virus titers (n = 3/group) harvested at 6 dpi. Lung virus loads are expressed as tissue culture infectious dose 50 (TCID50) per gram of lung extract. Individual virus titers are given and lines represent averages. Lung virus titers on the X axis are below detection limit. “M2e” in the X-axis means M2e-VLP.

Immunization of laboratory mice with M2e-VLP can protect against challenge with influenza A viruses regardless of the viral subtype^13^,^22^,^43^. Although the mechanism of protection afforded by M2e-based vaccination strategies largely relies on humoral immunity, antibodies against M2e have little if any direct impact on virus replication *in vitro* and their protection relies on Fcγ Receptors expressed by innate immune cells^44^. In contrast, antibodies directed against HA or NA, especially those with HAI or NAI activity, directly impact virus replication *in vitro*. We therefore set up a second vaccination-challenge experiment in which we compared immune protection induced by M2e, NA, HA and combinations thereof against homologous and heterologous influenza A virus challenge (Fig. 4a). This experiment allowed us to compare immune protection induced by vaccination with M2e-VLPs with protection induced by tetNA against H1N1v challenge. In addition, we evaluated if vaccination with tetNA or triHA + tetNA could benefit from co-administration of M2e-VLPs (Fig. 4a).

Inclusion of triHA in the vaccine resulted in seroconversion to HA as measured by ELISA (Fig. 4b) and only this group displayed HAI titers (Fig. 4e). Again, mice that had been vaccinated with a tetNA-containing formulation seroconverted to triHA based on ELISA, most likely due to antibodies directed to the shared Streptag (Fig. 4b). Anti-NA IgG serum responses and NAI titers were comparable in the three groups that had been immunized with a tetNA containing vaccine (Fig. 4c,f). M2e-specific serum IgG titers were comparable in all three groups that had been immunized with M2e-VLP-containing formulations (Fig. 4d). Finally, sera from mice that had been vaccinated with tetNA-containing antigens displayed H1N1v microneutralizing activity, with the highest activity observed in the group that had triHA included in the vaccine (Fig. 4g). The NAI and microneutralization titers were comparable in the tetNA only vaccinated groups between the two experiments (compare Figs 3e,f and 4f,g).

Half of the mice in each group were challenged with a sublethal dose (0.1LD_50_) of H1N1v. PBS and VLP immunized mice suffered body weight loss after challenge, which was significantly higher than in vaccinated animals (Fig. 4h). Mice that were vaccinated with tetNA + M2e-VLP + triHA displayed no body weight loss after H1N1v challenge (Fig. 4h). The M2e-VLP immunized mice lost up to 10% of their original body weight (Fig. 4h). We note that M2e in the M2e-VLP differs at four positions compared with the H1N1v challenge virus. Mice that were immunized with tetNA displayed very moderate body weight loss upon H1N1v challenge whereas mice that were immunized with both NA and M2e did not display body weight loss. Lung virus loads in the PBS and control VLP group reached 10^5^ TCID_50_ on day 6 after challenge, whereas challenged tetNA and tetNA + M2e-VLP + triHA vaccinated mice did not carry residual virus anymore at this time point (Fig. 4i). In line with previous reports, M2e-immune mice had considerable lung viral loads after challenge, although these were typically tenfold lower compared to the PBS and control VLP groups. Unexpectedly, two out of three mice that received tetNA + M2e-VLP had residual pulmonary virus on day 6 post infection (Fig. 4i). Taken together, triHA- and tetNA- containing vaccines provide excellent protection against homologous challenge. In addition, challenged M2e-VLP immunized mice are significantly protected against disease compared to the PBS- and control VLP-immunized mice.

### NA- and M2e-immune hosts mount functional humoral and T cell responses upon virus exposure when they lack HA-specific immunity

**Figure 5:**
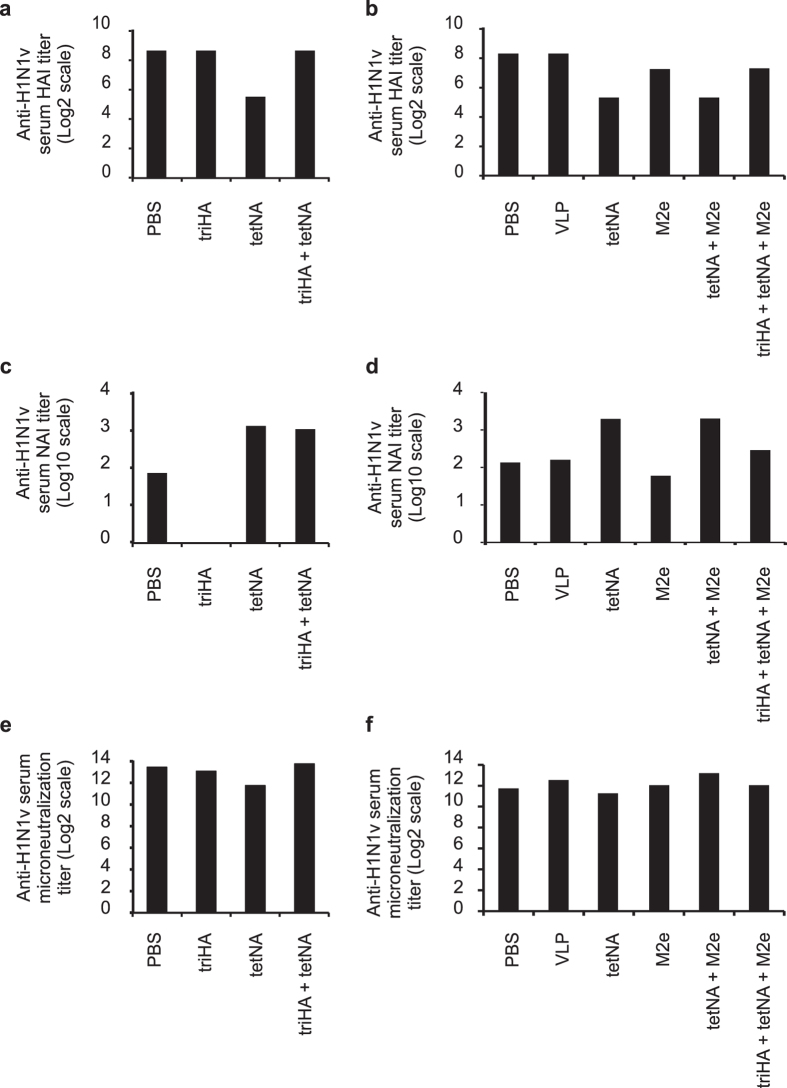
Infection-permissive immunity allows induction of humoral responses upon infection. Twenty one days after challenge with 0.1LD_50_ of H1N1v of BALB/c mice that had been vaccinated with the indicated antigens, pooled serum (n = 10) was used for *in vitro* antiviral assays. Results for experiment 1 are shown on the left and results for experiment 2 on the right. (**a,b**) HAI titers, (**c,d**) neuraminidase inhibiting titers and (**e,f**) microneutralization titers determined against H1N1v virus. “M2e” in the X-axis means M2e-VLP.

In the experiments described above we had created both infection-permissive (vaccination with tetNA, M2e-VLP and tetNA + M2e-VLP) and sterilizing immunity (vaccination with triHA, triHA + tetNA, and triHA + tetNA + M2e-VLP). We therefore assessed how these different types of pre-existing immune status would modulate the immune responses during and after recovery from H1N1v challenge. HAI titers against H1N1v were detectable in all mice after challenge (Fig. 5a,b). Pre- and post-challenge HAI titers were the same in mice that had been immunized with triHA-containing antigen formulations. All except the triHA only immunized mice had serum NAI titers after challenge and NAI titers were highest in challenged mice that had been immunized with a tetNA-containing vaccine (Fig. 5c,d). The absence of NAI in the triHA group and the lack of a boost of the pre-existing NAI titer in the triHA + tetNA + M2e-VLP groups, is in line with the sterilizing immunity induced by triHA in these vaccine compositions. Finally, all mice had significant and comparable H1N1v microneutralizing antibody titers after challenge (Fig. 5e,f).

**Figure 6:**
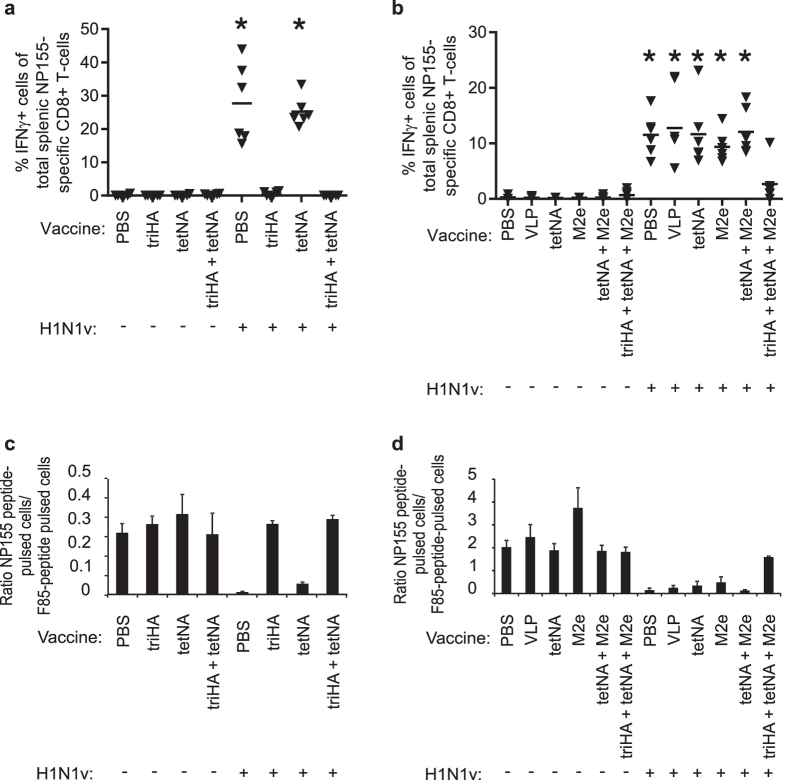
Infection-permissive immunity allows induction of cellular responses upon infection. Results for experiment 1 are shown on the left and results for experiment 2 on the right. (**a,b**) Ten days after mock challenge or challenge with 0.1LD_50_ of H1N1v of BALB/c mice that had been vaccinated with the indicated antigens, 6 mice from each group were sacrificed and splenocytes were isolated. Splenocytes were restimulated *ex vivo* with NP155 peptide for 6 hours. IFN-γ production in NP-specific CD8+ T cells was determined by flow cytometric analysis by intracellular cytokine staining of NP155 pentamer positive cells. *P value < 0.01 compared to respective mock-infected groups with two-sided Mann-Whitney *U* test with correction for ties. The horizontal line denotes the median. (**c,d**) *In vivo* clearance of NP155-pulsed target cells. Splenocytes from naïve mice were pulsed *in vitro* for 1 h with NP155 or irrelevant Respiratorial Syncytial Virus-derived F85 peptide, differentially labeled with CFSE and injected intravenously in mice 9 days after mock or H1N1v challenge. Nineteen hours later, pulsed target cells were recovered from the spleens of the recipient mice and analyzed by flow cytometry. Bars represent averages (n = 3/group) and error bars represent standard deviations. NP155-pulsed and F85-pulsed target cells were labeled with 1 μM CFSE and 10 μM CFSE respectively in (**c**) and with 10 μM CFSE and 1 μM CFSE respectively in (**d**). Higher concentrations of CFSE result in lower cell recovery which explains tenfold higher ratios in (**d**) compared to (**c**). “M2e” in the X-axis means M2e-VLP.

We next quantified CD8+ T cell responses specific for the cross-reactive NP T cell epitope TYQRTRALV (NP155) in splenocytes isolated on day 10 after mock or H1N1v challenge. Importantly, NP was only introduced in the animals by the H1N1v challenge virus, not by any of the recombinant vaccine antigens. We observed significant levels of interferon gamma (IFNγ) producing, NP155-specific CD8+ T cells in all challenged mice except for the groups that had been immunized with triHA containing vaccine: *i.e.* triHA and triHA + tetNA in experiment 1 and triHA + tetNA + M2e-VLP in experiment 2 (Fig. 6a,b; p < 0.01 compared to respective mock-infected groups). Although vaccination with infection-permissive vaccine antigens protected mice against morbidity, NP155-specific T cell responses in these mice were comparable in magnitude with those observed in unprotected PBS or control VLP immunized mice. To assess the functional capacity of these T cells we performed an *in vivo* killing assay. CFSE-labeled NP155-peptide pulsed target cells were injected into convalescent or mock challenged mice and these cells were retrieved 19 h later from the spleen and quantified by flow cytometry. All challenged mice, except those that had been immunized with triHA-containing vaccine, exhibited specific target cell killing (Fig. 6c,d). Taken together, pre-existing HAI immunity protects against a homologous challenge and strongly dampens the NP-specific T cell response upon challenge. In contrast, immunization with tetNA and M2e-VLP, separate or combined, provides near complete immune protection against homologous challenge without affecting the NP-specific CD8+ T cell response.

### Cytokine and chemokine milieu correlates with lung virus loads

**Figure 7:**
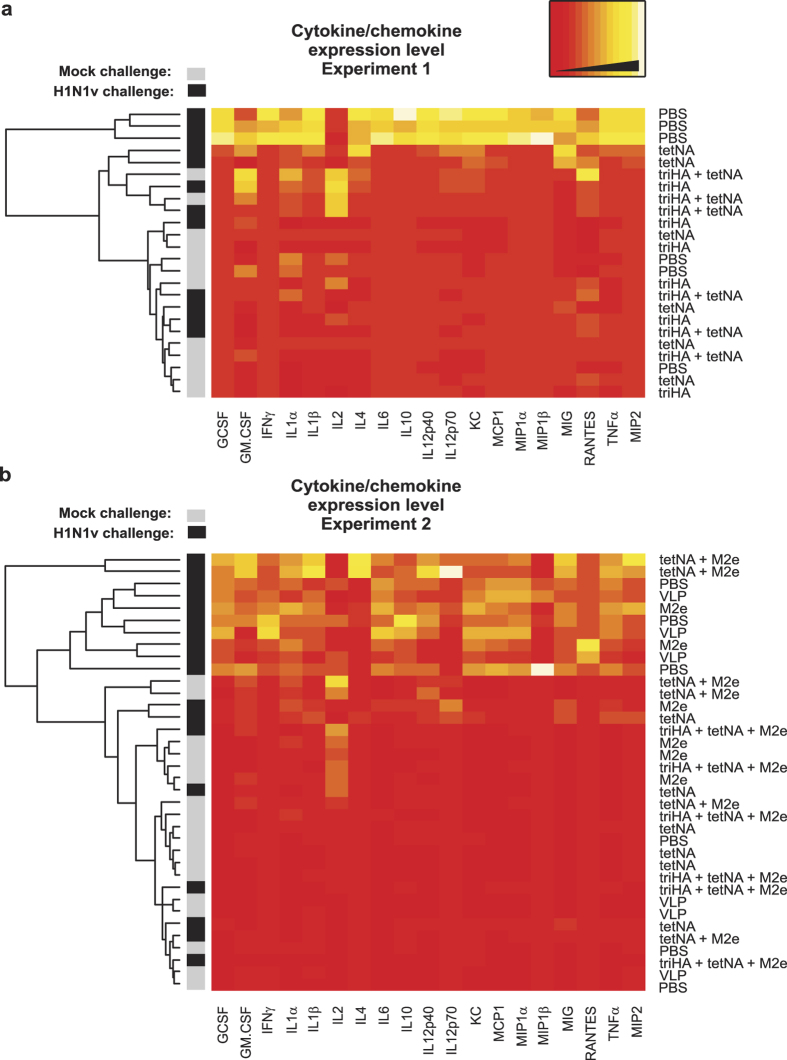
Heat maps representing cytokine and chemokine concentrations measured in cleared lung homogenates from individual mice (n = 3/group) harvested at 6 days after mock infection or infection with 0.1LD_50_ of H1N1v for experiment 1 and 2. Cytokine and chemokine expression levels were determined using a 19-plex cytokine bead array. Each column represents a specific cytokine or chemokine as indicated at the bottom of the heat map. Each row represents an individual mouse, with the vaccine status indicated on the right hand side of the heat map. Individual samples were clustered and rows were reordered based on the profile of cytokine and chemokine expression. Infection status and hierarchical cluster dendrogram is shown at the left hand side of the heat map. “M2e” on the right hand side of the heat map means M2e-VLP.

Replicating virus can cause the release of a plethora of cytokines and chemokines, derived from directly infected cells and from recruited immune cells. We quantified 19 chemokines and cytokines in lung samples isolated on day six after challenge with H1N1v. Overall, the extent of virus replication (Figs 3h and 4i) positively correlated with the levels of pro-inflammatory cytokines and chemokines measured in the lung samples (Fig. 7a and Supplementary Fig. 1 for experiment 1, Fig. 7b and Supplementary Fig. 2 for experiment 2). For example, triHA-immunized mice, which had undetectable pulmonary virus titers in the lungs had cytokine and chemokine levels that were comparable to those in mock-challenged mice. TetNA-immunized mice controlled H1N1v replication better than M2e-immune mice, and in general their cytokine and chemokine expression patterns are also similar to those of mock-infected animals. Unexpectedly, two out of three mice that had been immunized with tetNA + M2e-VLP showed a pro-inflammatory cytokine and chemokine signature that was comparable to that of challenged, unprotected PBS and control VLP groups (Fig. 7b). In particular IL4 levels were high in these samples (Fig. 7b) (Supplementary Fig. 2).

### Pulmonary germinal center formation after H1N1v infection

**Figure 8:**
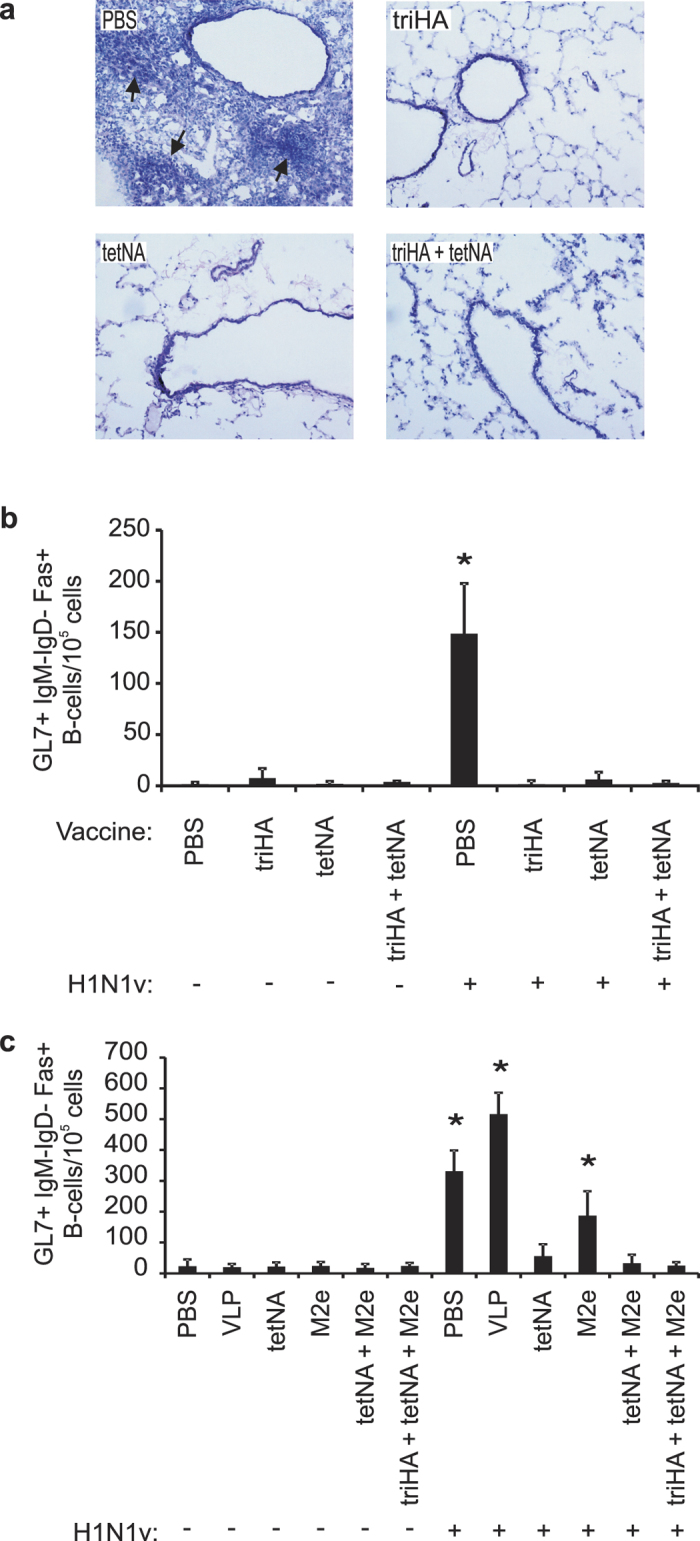
Formation of inducible bronchus associated lymphoid tissue (iBALT) after primary challenge with 0.1LD_50_ of H1N1v is observed in M2e-immune but not in HA- or NA-immune mice. (**a**) Representative lung sections isolated on day 21 after challenge of mice with 0.1 LD_50_ of H1N1v. Lung sections were stained with hematoxylin/eosin to illustrate presence or absence of possible iBALT structures (arrows) near bronchi in lungs of infected mice (10× magnification). Data are from experiment 1 with the vaccination status indicated as insets. (**b**) Presence of iBALT at 21 dpi (mock or H1N1v) was investigated by quantification of GL7+ immunoglobulin (Ig)M-IgD-Fas+ B-cells in total lung cells by flow cytometry. PBS mice had significantly more GL7 + IgM-IgD-Fas+ B-cells compared to mock-challenged controls (Two-sided Mann-Whitney *U* test with correction for ties, *P value < 0.05). Bars represent averages of four mice, error bars represent standard deviations. Data are from experiment 1 with the vaccination and challenge status of the mice indicated below the X-axis. (**c**) Presence of iBALT at 21 dpi (mock or H1N1v) was investigated by quantification of GL7 + IgM-IgD-Fas+ B-cells in total lung cells by flow cytometry. PBS, VLP and M2e-VLP mice had significantly more GL7 + IgM-IgD-Fas+ B-cells compared to mock-challenged controls (Two-sided Mann-Whitney *U* test with correction for ties, *P value < 0.05). Bars represent averages of four mice, error bars represent standard deviations. Data are from experiment 2 with the vaccination and challenge status of the mice indicated below the X-axis.

Germinal center formation is induced in lungs of naïve mice that survive an influenza A virus infection and is a key feature of inducible bronchus-associated lymphoid tissue (iBALT)^45^,^46^. Therefore, histopathological analysis of lung section from mice that had been vaccinated and challenged in experiment 1 was performed to evaluate the extent of immune infiltration and potential iBALT formation. iBALT typically consists of structured areas with DCs, T and B cells at peribronchial and perivascular locations in the lung^45^. Lung sections from PBS vaccine control mice analyzed on day 21 after challenge, displayed such dense peribronchial structures containing infiltrating immune cells, indicative for the presence of iBALT (Fig. 8a, arrows). In contrast, challenged mice that had been vaccinated with triHA, tetNA or a combination of both, lacked these dense peribronchial structures (Fig. 8a).

We also determined GL7 expression, a feature of activated B and T cells involved in germinal center formation^47^ by flow cytometry to compare the extent of germinal center formation. In line with our previous findings^13^, we observed that 21 days after infection, the highest number of GL7 + IgM-IgD-Fas+ B-cells was induced in the control mice that had received PBS or control VLP antigen (Fig. 8b,c). Challenged M2e-VLP immunized mice also had a significantly higher number of GL7 + IgM-IgD-Fas+ B cells in the lungs compared to mock challenged controls (Fig. 8c). In contrast, GL7 + IgM-IgD-Fas+ B cells remained close to baseline after H1N1v challenge of mice immunized with triHA- or tetNA-containing vaccines (Fig. 8b,c).

### Lack of heterosubtypic immunity induced by prior infection in triHA-immunized mice

**Figure 9:**
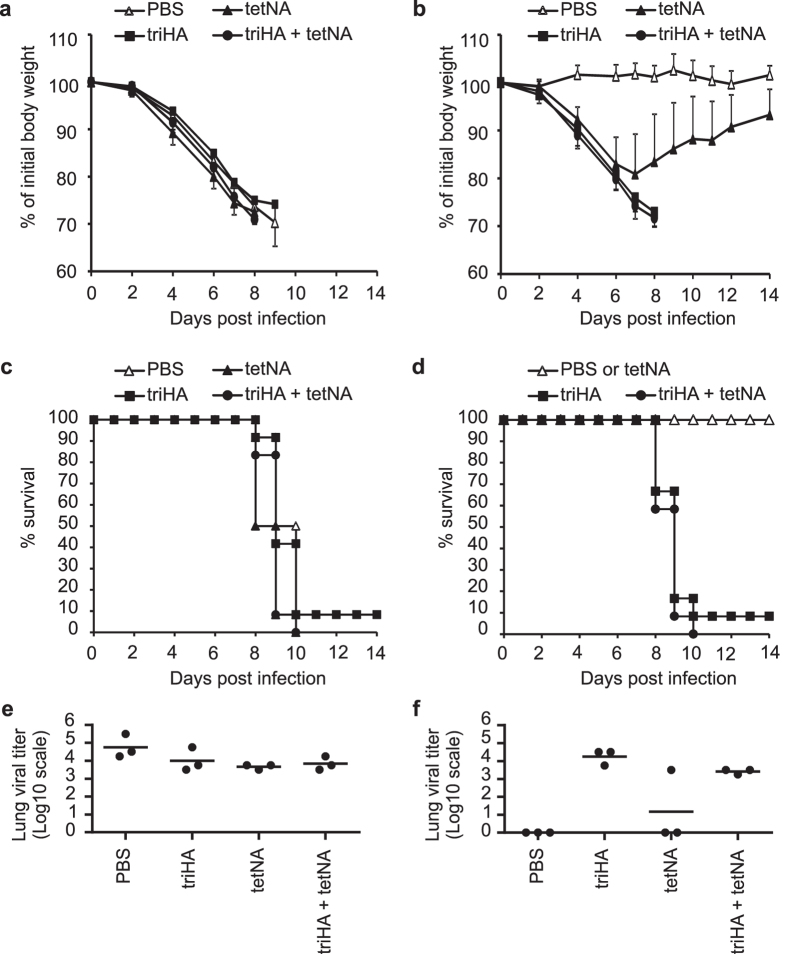
Prior infection with H1N1v virus contributes to protection against subsequent infection with heterologous H3N2 virus. Data are from experiment 1 with the vaccination status indicated. (**a**) Weight loss following challenge with H3N2 virus (2LD_50_) four weeks after mock challenge of mice vaccinated as indicated (n = 12/group). (**b**) Weight loss of mice vaccinated as indicated following secondary challenge with H3N2 virus (2LD_50_) four weeks after primary sublethal challenge with H1N1v (n = 12/group). Compared to PBS mice, body weight loss was significantly higher in triHA and in triHA + tetNA mice between days 2 and 8 after infection, and in tetNA mice between days 4 and 14 after infection (Two-sided Mann-Whitney *U* test with correction for ties, P value < 0.05). (**c**) Mortality of mice vaccinated as indicated following challenge with H3N2 virus (2LD_50_) four weeks after mock challenge or (**d**) four weeks after primary sublethal challenge with H1N1v (n = 12/group). (**e,f**) Lung virus titers in cleared lung homogenates harvested at 6 dpi with H3N2 virus (2LD_50_) of mice vaccinated as indicated (n = 3/group). H3N2 challenge was given four weeks after mock challenge (**e**) or four weeks after primary sublethal challenge with H1N1v (**f**). In (**e,f**) individual virus titers are given and lines represent averages. Lung virus loads are expressed as tissue culture infectious dose 50 (TCID_50_) per gram of lung extract. Lung virus titers on the X axis are below detection limit.

**Figure 10:**
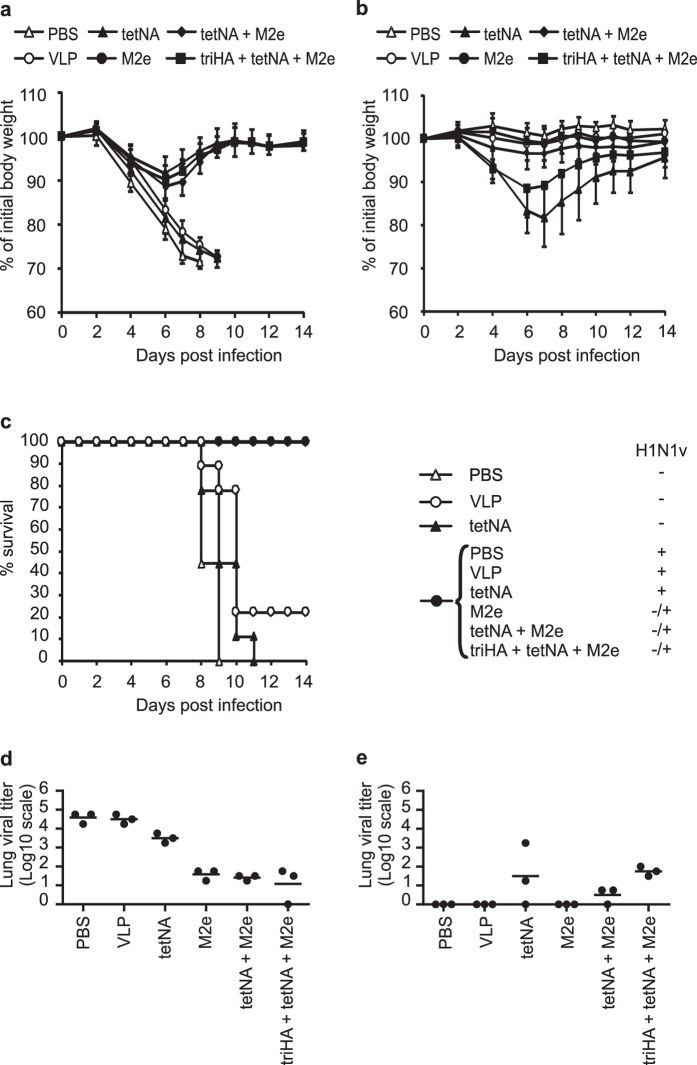
Prior infection with H1N1v virus contributes to protection against subsequent infection with H3N2 virus. Data are from experiment 2. (**a**) Weight loss following challenge with H3N2 virus (2LD_50_) four weeks after mock challenge (n = 9/group). Compared to PBS mice, body weight loss was significantly different at 4 dpi and 7 dpi in mice vaccinated with tetNA (P < 0.05), and between 4–8 dpi in mice vaccinated with tetNA + M2e-VLP and triHA + tetNA + M2e-VLP (P < 0.01). Compared to VLP mice, body weight loss was significantly different between 6–9 dpi in M2e-VLP, tetNA + M2e-VLP and triHA + tetNA + M2e-VLP mice (P < 0.05). Compared to tetNA mice, body weight loss was significantly different between 6–9 dpi in mice vaccinated with tetNA + M2e-VLP or triHA + tetNA + M2e-VLP (P < 0.01). (**b**) Weight loss of mice vaccinated as indicated following secondary challenge with H3N2 virus (2LD_50_) four weeks after primary sublethal challenge with H1N1v (n = 9/group). Compared to PBS mice, body weight loss was significantly higher between 4–14 dpi in mice vaccinated with tetNA, tetNA + M2e-VLP and triHA + tetNA + M2e-VLP (P < 0.05). Compared to VLP mice, body weight loss was significantly higher at 4 dpi in mice vaccinated with tetNA + M2e-VLP (P < 0.05) and between 4–10 dpi and at 14 dpi in mice vaccinated with triHA + tetNA + M2e-VLP (P < 0.05). Mice vaccinated with tetNA showed significantly more body weight loss between 4–14 dpi compared to tetNA + M2e-VLP mice (P < 0.05) and at 7 dpi compared to triHA + tetNA + M2e-VLP mice (P < 0.05). (**c**) Mortality of mice vaccinated as indicated following challenge with H3N2 virus (2LD_50_) four weeks after mock challenge or primary sublethal challenge with H1N1v (n = 9/group). (**d**) Lung virus titers (n = 3/group) in cleared lung homogenates harvested at 6 dpi of mice vaccinated as indicated following challenge with H3N2 virus four weeks after mock challenge or (**e**) after primary sublethal challenge with H1N1v. In (**e**,**f**) individual virus titers are given and lines represent averages. Lung virus loads are expressed as TCID_50_ per gram of lung extract. Lung virus titers on the X axis are below detection limit. “M2e” in the X-axis means M2e-VLP. Statistical analysis was by two-sided Mann-Whitney *U* test with correction for ties.

Our sequential infection model allowed to evaluate how the different vaccine regimens would perform against primary heterosubtypic challenge (mock followed by H3N2), and how vaccination followed by challenge with H1N1v would potentially influence protection against a subsequent challenge with a heterosubtypic influenza virus (H1N1v followed by H3N2)(Fig. 3a). All remaining mice from experiment 1 and 2 were thus challenged on day 70 with 2 LD_50_ of a mouse-adapted H3N2 virus (i.e. seven weeks after the booster immunization). Mice that had been immunized with triHA or tetNA, separate or combined, and mock challenged on day 42 were not protected against H3N2 challenge and displayed morbidity and mortality that was comparable to PBS and control VLP immunized mice (Figs 9a,c and 10a,c). This illustrates that vaccination with H1N1 triHA and tetNA (separate or combined) does not protect against challenge with an H3N2 virus. In contrast, all mock-infected mice that had been immunized with M2e-VLP containing vaccines, separately or combined with triHA or tetNA survived the H3N2 challenge but experienced transient body weight loss (Fig. 10a,c). This outcome illustrates the universal influenza A protection offered by M2e-based vaccination and also its capacity to control but not completely prevent disease following challenge. When mice that had been previously exposed to H1N1v were rechallenged with H3N2 virus, the outcome was strikingly different. PBS and control VLP immunized mice that had recovered from prior H1N1v challenge were now fully protected against the H3N2 challenge (Figs 9b,d and 10b,c). In contrast, triHA, or triHA + tetNA vaccinated mice were not protected against the rechallenge with H3N2 virus (Fig. 9b,d). TetNA only immunized mice3 survived the rechallenge with H3N2 virus but experienced significant body weight loss (Figs 9b,d and 10b,c). All mice that had been immunized with M2e-VLP and then challenged with H1N1v survived the secondary H3N2 challenge and did not display any body weight loss. This illustrates that in the latter group the protection against the H3N2 challenge was at least partially mediated by the H1N1v elicited immune response and not solely the consequence of anti-M2e antibodies. In the triHA + tetNA+ M2e-VLP immunized mice morbidity after rechallenge with H3N2 was highest and comparable to the body weight loss seen in mice that had been vaccinated similarly but for which the H3N2 virus was the first challenge (Fig. 10a,b). This suggests that there was no contributing protection from the prior H1N1v challenge against secondary challenge in the triHA + tetNA + M2e-VLP group and that protection was largely if not exclusively derived from M2e-specific immunity induced by the vaccination.

We also determined the H3N2 lung virus load following mock/H3N2 and H1N1v/H3N2 challenge. In the mock/H3N2 groups, only M2e-VLP immunized mice displayed significantly reduced lung virus titers compared with PBS and control VLP immunized mice (Figs 9e and 10d). NA-immune mice showed a modest reduction in virus titers upon heterologous H3N2 challenge without prior H1N1v challenge, but this was noticed only in the second of the 2 experiments (Figs 9e and 10d). In the groups challenged with H1N1v followed by H3N2, no residual lung virus was detectable on day six after secondary challenge with H3N2 virus in PBS, control VLP and M2e-VLP immunized mice (Figs 9f and 10e). One out of three (experiment 1) and two out of three (experiment 2) tetNA immunized mice had residual H3N2 virus on day 6 after rechallenge (Figs 9f and 10e). The triHA and triHA + tetNA immunized mice had the highest lung virus titers after rechallenge with H3N2 virus (Fig. 9f). Taken together, M2e-VLP immunization provides broad and disease-modulating immunity against influenza A virus infection. Supplementing tetNA antigen with M2e-VLP improves the protection against primary homologous and secondary heterosubtypic infection compared to tetNA alone. Finally, immunization with H1N1v-derived triHA vaccine with or without added tetNA antigen does not protect against primary (mock/H3N2) or secondary (H1N1v/H3N2) challenge with a heterosubtypic virus in the absence of M2e-specific immunity.

### T cell responses induced by primary homologous infection are boosted after secondary heterologous challenge

**Figure 11:**
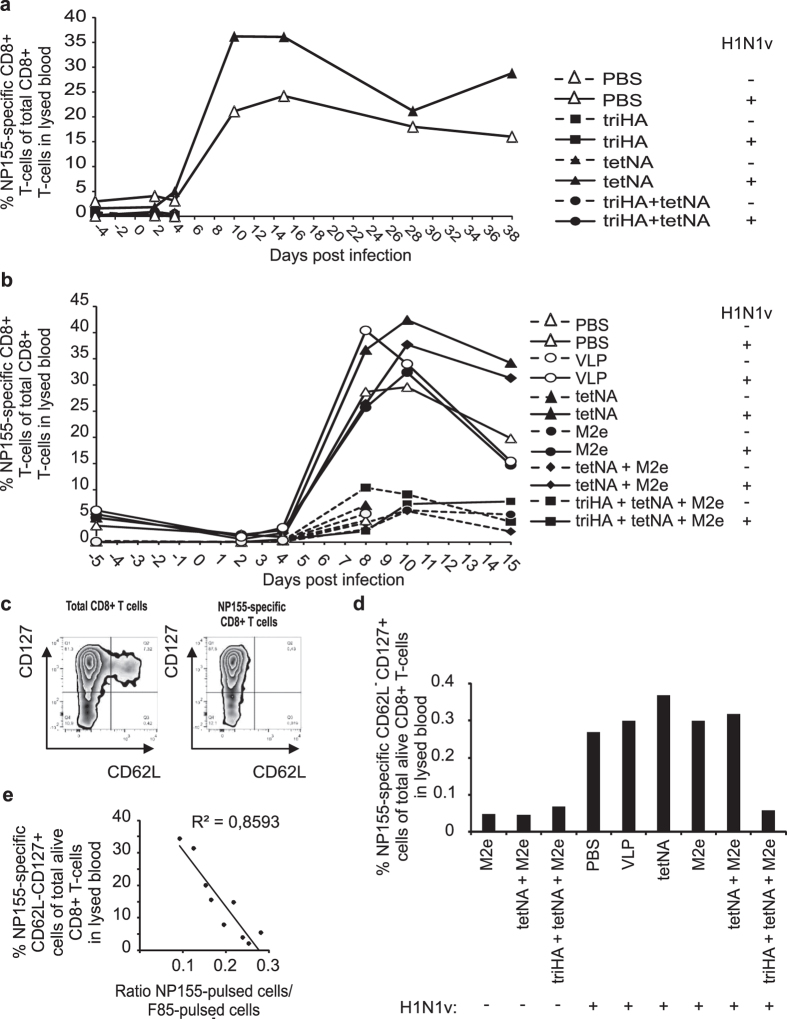
Mice with NP-specific T-cells in circulation show higher and faster T cell responses upon secondary infection with heterologous virus. (**a**) Kinetic analysis of NP-specific CD8+ T cell frequencies before and after challenge with H3N2 virus (2LD_50_) by flow cytometry following NP155 pentamer staining on whole lysed blood of pools (n = 3/group). Data are from experiment 1 and the vaccination and prior mock or H1N1v challenge status of the mice are indicated on the right. Only PBS and tetNA vaccinated mice that had experienced prior H1N1v challenge survived the H3N2 challenge. (**b**) Kinetic analysis of NP-specific CD8+ T cell frequencies before and after challenge with H3N2 virus (2LD_50_) by flow cytometry following NP155 pentamer staining on whole lysed blood of pools (n = 3/group). Data are from experiment 2 and the vaccination and prior mock or H1N1v challenge status of the mice are indicated on the right. “M2e” means M2e-VLP. Only PBS and tetNA vaccinated mice that had experienced prior H1N1v challenge or mice vaccinated with M2e-VLP survived the H3N2 challenge. PBS-, VLP- and tetNA-vaccinated mice that had previously been mock-challenged did not survive the H3N2 challenge. (**c**) Representative density plots for cell surface markers CD127 and CD62L on total (left plot) and NP-specific (right plot) circulating CD8+ T cells. (**d**) Frequencies of NP-specific CD62L- CD127+ CD8+ T cells in whole lysed blood of pools (n = 3/group) collected on 10 dpi. “M2e” in the X-axis means M2e-VLP. (**e**) Correlation between frequency of NP-specific CD62L- CD127+ CD8+ T cells in whole lysed blood of pools (n = 3/group) collected at 10 dpi and *in vivo* clearance of NP155 peptide-pulsed target cells recovered from spleens 6 h after intravenous administration of these cells. Data in c-e are from experiment 2.

Finally, we determined the NP-specific CD8+ T cells at different time points after mock/H3N2 and H1N1v/H3N2 challenge. NP-specific T cells were determined in lysed whole blood collected before and at different time points after H3N2 challenge. Mice that had been mock challenged as well as mice that had received triHA (triHA, triHA + tetNA and triHA + tetNA + M2e-VLP) followed by challenge with H1N1v did not have detectable NP-specific CD8+ T cells before H3N2 challenge (Fig. 11a,b). In the blood of PBS, control VLP, tetNA-, M2e-VLP- and tetNA + M2e-VLP-vaccinated mice that had been challenged before with H1N1v, NP155-specific CD8+ T cells were detectable prior to rechallenge with H3N2 virus (Fig. 11a,b). In these mice, the percentage of NP155 pentamer positive T cells dropped during the first days after H3N2 challenge, but increased drastically between day 4 and 8 after the H3N2 infection. This increase in percentage of NP-specific CD8+ T cells was higher and faster compared to that in mice that were previously mock-challenged or in H1N1v/H3N2 mice that had received triHA (triHA, triHA + tetNA or triHA + tetNA + M2e-VLP groups). NP-specific CD8+ T cell levels remained highest over time in NA only-immune mice that had been challenged earlier with H1N1v virus.

In mice from experiment 2, we also stained the surface markers CD127 and CD62L on NP-specific CD8+ T cells in lysed whole blood collected at 10 days post infection with H3N2 virus. CD127 is the receptor for IL 7 and is expressed on memory precursor cells early after infection^48^ whereas CD62L is a lymph node homing receptor, which allows discrimination between effector and central memory T cells^49^. Although over 7% of total memory (CD127+) CD8+ T cells in the blood showed surface expression of CD62L, NP155-specific CD8+ T cells were negative for CD62L, indicating their effector memory nature (Fig. 11c). Mice that had been vaccinated with tetNA or tetNA + M2e-VLP followed by challenge with pandemic H1N1v and then H3N2 virus showed the highest percentage of CD127+ CD62L- cells (Fig. 11d). M2e-VLP vaccinated and control mice had somewhat lower frequencies of CD127+ CD62L- NP-specific CD8+ T cells than tetNA-only vaccinated mice. In the triHA + tetNA + M2e-VLP immunized group, frequencies of CD127+ CD62L- NP-specific CD8+ T cells were comparable to those in mice that had been mock-challenged before. To investigate the activity of the induced NP-specific T cells we performed an *in vivo* cell killing assay. In parallel the frequency of circulating CD127+ CD62L- NP-specific effector memory CD8+ T cells was measured as described above. Figure 11e illustrates that the frequencies of CD127+ CD62L- NP-specific CD8+ T cells in circulation correlated with clearance of NP155-peptide pulsed target cells *in vivo*.

We conclude that NP-specific CD8+ T cells are still circulating four weeks after primary H1N1v challenge in mice that lacked HAI immunity provided by vaccination. Furthermore, this pre-existing T cell pool expanded quickly upon rechallenge with heterologous H3N2 virus. Importantly whereas vaccination with HA (triHA + tetNA + M2e-VLP) prevented the induction and boosting of cross-protective cell killing T cells, M2e and/or tetNA vaccination allowed induction and boosting of these cells.

## Discussion

We compared seven different vaccination regimens ranging from none (PBS and control VLP) to complete (triHA + tetNA + M2e-VLP) membrane-exposed viral antigen coverage in the recombinant vaccine antigens. Our findings reveal that NA- and M2e-based vaccination strategies, in the absence of HA immunity, can offer significant immune protection without compromising the induction of cross-reactive CD8+ T cells upon influenza A virus infection. We showed that vaccination with recombinant soluble oligomeric forms of HA and NA and to a lesser extent with M2e-VLPs, protected against morbidity following challenge with a sublethal dose of homologous H1N1v virus. Therefore, increasing and standardizing the content of NA in conventional influenza vaccines could improve protection in cases where HA-based protection is reduced due to antigenic mismatch with circulating virus strains or in individuals that mount a suboptimal response against currently licensed flu vaccines.

Only immunization with M2e-VLPs protected against primary challenge with heterosubtypic H3N2 virus. M2e-based vaccine candidates have not been evaluated in human beyond phase 1 clinical trials. Nevertheless, a study in human volunteers in which a M2e-specific human monoclonal antibody was administered 24 hours after a controlled challenge with A/Wisconsin/67/2005 (H3N2) virus, significantly reduced infection related symptoms, suggesting that M2e-based vaccines could contribute to protection against influenza A^50^.

Currently licensed inactivated influenza vaccines mainly aim at the induction of HA-specific, virus-neutralizing immunity, which necessitates almost yearly vaccine updates due to antigenic drift and shift in circulating viruses. NA of human influenza viruses is also subject to antigenic drift^34^, which suggests that like HA, the NA molecule is under immune selection pressure. Despite being recognized as a good vaccine candidate for several decades^21^ and having shown to be safe and immunogenic when administered unadjuvanted to humans^51^, the NA protein content is neglected in influenza vaccines. Due to its higher representation on the viral surface, HA is considered to be immunodominant over NA in the intact virus^52^. When administered together at equal amounts as recombinant soluble molecules formulated in oil-in-water SAS adjuvant, we also observed a trend of immunodominance of triHA over tetNA. We found that triHA was rapidly desialylated in the presence of tetNA, even when the two glycoproteins were co-incubated at 4 degrees Celsius for a short time (Fig. 1f). In the case that the antigens were combined, triHA and tetNA were coincubated under similar conditions prior to injection into mice, and triHA was thus likely delivered as a desialylated protein in those groups. Although it has been proposed that the extend of N-glycosylation of HA may influence the B cell response after vaccination^53^, we did not observe a difference in ELISA, HAI and microneutralization titer between triHA and triHA + tetNA vaccinated groups (Fig. 3b,d,f). In addition, triHA and triHA + tetNA vaccinated mice were equally sensitive to primary H3N2 infection as tetNA vaccinated mice. This suggests that desialylation of HA did also not enable significant induction of cross-protective antibodies that can protect against both group 1 and group 2 influenza virus infections.

NA-based immunity can protect mice against challenge with homologous influenza virus^21^,^30^. Likewise, we found that vaccination with tetNA protected from severe virus-induced morbidity following H1N1v challenge. However, complete absence of morbidity was only obtained by supplementing tetNA with M2e-VLP and triHA. M2e-immune mice showed a reduction in lung viral load compared to PBS and control VLP groups, but NA-immunity performed much better for this parameter. This is most likely due to the fact that M2e-specific antibodies likely protect by eliminating infected cells through Fcγ receptor-dependent mechanisms^44^ rather than by binding to influenza virions^13^,^21^. On day 6 after challenge there was no virus detectable in lung homogenates of NA-immune mice (Figs 3h and 4i). This suggests that replicating virus was already cleared in NA-immune mice by that time point as opposed to sterilizing immunity provided by HA vaccination, which resulted in undetectable virus at all time points in our mouse model (Fig. 2c). In naïve mice influenza virus infection induces cross-reactive T cell responses that provide protection against challenge with influenza virus of a different subtype^4^,^54^,^55^. Comparable NP155-specific T cell responses were measured in spleens of PBS, control VLP, M2e-VLP and tetNA immunized mice after challenge with H1N1v. This suggests that the reduced viral replication in tetNA mice does not lead to limiting concentrations of viral antigen available for immune presentation and T cell priming and/or that formation of NA-specific antibody-antigen complexes in the course of infection contributes to viral antigen presentation to T cells.

Mice vaccinated with M2e-VLP alone or in combination with tetNA and/or triHA were protected from heterologous challenge by the universal M2e-based vaccine. However, heterologous protection was enhanced in M2e-immune mice that had mounted NP-specific CD8+ T cells during a prior infection (Fig. 10a,b). This highlights the advantage of infection-permissive over virus-neutralizing immunity in this sequential influenza infection model^13^,^56^ and the correlation of cross-reactive T cells with heterosubtypic immunity^13^. However, we only analyzed T cell responses in the spleen for one well characterized CD8+ T cell epitope and at one time point (day 10) after the H1N1v challenge. It is possible that T cell responses to other viral epitopes, dominant or subdominant, as well as recall responses after a second heterosubtypic virus challenge, were affected by NA-immunity.

Mice with NA-specific immunity did not display iBALT following H1N1v infection (Fig. 8). These tertiary lymphoid organs have also been observed in humans^57^ and are marked by specific structural organization and formation of germinal center reactions^13^,^46^. The observation that M2e vaccinated but not tetNA vaccinated mice develop iBALT could be explained by the higher or longer viral replication in M2e vaccinated mice (Fig. 4i). Inducible BALT also contributes to heterosubtypic immunity^45^, which may explain why protection against a second challenge with H3N2 virus was stronger in PBS- and VLP- than in tetNA-vaccinated mice despite similar T cell levels in the spleen. Our data suggest that induction of iBALT requires a certain degree of virus replication, with the associated production of cytokines and chemokines and morbidity. Indeed, local production of chemokines like CCL19, CCL21 and lymphotoxin β is important for iBALT formation^46^,^58^,^59^. The difference in infection-permissivity provided by M2e− or NA-based vaccines results in different cytokine and chemokine profiles (Fig. 7) and may therefore influence iBALT formation.

NP-specific CD8+ T cells remained detectable in lysed blood until four weeks after challenge with H1N1v in NA- and M2e-immune mice. This suggests that infection-permissive immunity provided by vaccination with M2e or NA antigens does not interfere with induction of memory T cell responses. Wiley *et al.* reported that virus-specific T cells peaked between days five to seven after rechallenge, depending on their location in the respiratory tract^54^. Therefore, it is possible that the actual peak of NP155-specific CD8+ T cells is between four and ten days after H3N2 challenge. During the retraction phase, frequencies of NP155-specific CD8+ T cells remained highest in H1N1v-experienced mice that had been immunized with tetNA or tetNA + M2e-VLP. These mice also had the highest frequencies of NP155-specific CD127+ T cells. CD127, the receptor for interleukin 7, is considered a marker for memory T cells that is expressed already early after infection^48^. Moreover, all NP155-specific CD8+ T cells that expressed CD127 were negative for the lymph node-homing marker CD62L, which classifies them as effector memory T cells. TetNA or tetNA + M2e-VLP mice displayed higher morbidity and had higher viral load after secondary heterologous infection with H3N2 virus compared to mice vaccinated with control vaccines or M2e-VLP only. The higher viral load after heterologous infection in tetNA and tetNA + M2e-VLP mice may have contributed to higher T cell responses, as more viral antigen was available for presentation and therefore for boosting the pre-existing CD8+ T cell response induced by prior infection with H1N1v. On the other hand we can’t exclude an immunomodulatory effect due to NA-specific immunity, which may result in more efficient boosting of pre-existing T cell responses.

Irrespective of prior encounter with virus, HA-immune mice (triHA, triHA + tetNA and triHA + tetNA + M2e-VLP) showed very low frequencies of NP-specific CD8+ T cells in circulation compared to those observed in mice that had not been exposed before to H1N1v virus. This illustrates that blunting virus-induced T cell responses during homologous virus challenge by providing strong HA-specific immunity results in failure to prime cross-reactive T cell responses. Caution should be taken however as T cell immunity against influenza is particularly strong in mice, compared to the human situation. Still, in the absence of neutralizing antibodies, T cells in human correlate with protection against disease caused by influenza A viruses^2^,^3^.

Our results highlight the advantages of infection-permissive vaccination as it allows induction of heterosubtypic immunity upon virus exposure. Interfering with virus replication by virus-neutralizing antibodies induced with conventional vaccines clearly is advantageous for specific at risk target groups like pregnant women, immunocompromised people and the elderly. Prioritizing influenza vaccination in young children will most probably protect them for one influenza season, and continue to do so each time the flu vaccine is re-administered to keep track with viral antigenic drift. However, such practice may also hamper building up of cross-reactive T cell responses, each time a child is exposed to influenza virus^11^. On the other hand, one should also bear in mind that in practice conventional vaccines may fail to induce virus-neutralizing antibodies to levels that completely neutralize virus in the respiratory tract, and therefore are to some extent infection-permissive. In addition, it would be worthwhile to investigate to which extent a mismatched vaccine, as was the case for the H3N2 component during the 2014–2015 season, is associated with the induction of cross-protective T cells in influenza virus infected vaccinees.

## Methods

### Influenza challenge viruses and mice

The A/Belgium/2009 pandemic H1N1 virus is derived from a clinical isolate kindly provided by Dr Isabelle Thomas (Scientific Institute of Public Health, Brussels, Belgium). The influenza A/X47 H3N2 virus is a reassortant laboratory strain (A/Victoria/3/75 (H3N2) × A/Puerto Rico/8/34 (H1N1)). Viruses were adapted to mice by serial passages and produced in Madin Darby Canine Kidney (MDCK) cells in serum-free medium in the presence of TPCK (L-1-tosylamide-2-phenylethyl cholormethyl ketone)-treated trypsin (Sigma-Aldrich). All infection experiments were performed in a biosafety level 2^+^-contained laboratory. The sequence of mouse-adapted A/Belgium/2009 pandemic virus has been determined and is available at http://www.ncbi.nlm.nih.gov/nuccore/?term=txid1502382.

Female BALB/c mice aged 6–8 weeks were obtained from Charles River and housed under specified pathogen-free conditions with food and water *ad libitum*. Mice were challenged intranasally with 50 μl virus preparation diluted in PBS under mild isoflurane anesthesia. Body weight loss was measured as a read-out for morbidity. Mice were killed and considered dead in the survival analysis when a decrease of >30% in body weight was observed. All experiments were approved by and performed according to the guidelines of the animal ethical committee of Ghent University. The methods used were carried out in accordance with the approved guidelines.

### Vaccine antigens and immunization

Trimeric soluble hemagglutinin triHA and tetrameric soluble NA (tetNA) were produced with an N-terminal CD5-derived secretion signal and a Strep tag for purification purposes (Fig. 1a). The trimeric conformation of HA was stabilized by C-terminal fusion to the trimeric variant of GCN4 leucine zipper^60^. The tetrameric conformation of NA was stabilized by N-terminal fusion to the tetrabrachion tetramerization coiled coil^61^. The HA and NA expression constructs were ordered as synthetic genes from Genescript and cloned into the pEF mammalian expression vector. HEK293T cells were cultured in Dulbecco’s Modified Eagle’s Medium supplemented with 5% of fetal calf serum, non-essential amino acids, glutamate and antibiotics and were transfected with the HA and NA expression vectors using calcium phosphate Culture supernatant was collected 96h after transfection and cleared by centrifugation and filtration (0.22 μm Steritop filter, Millipore). Recombinant soluble triHA and tetNA was trapped on a 5 ml StrepTrap HP column (GE Healthcare Life Sciences) and eluted with 2.5 mM desthiobiotin (Sigma-Aldrich) in PBS using an AKTApurifier purification system (GE Healthcare Life Sciences). Affinity purification was followed by a polishing step with size exclusion chromatography (65 cm Superdex 200 for triHA and 30 cm Superdex 200 column for tetNA, GE Healthcare Life Sciences). NA activity was determined by liberation of 4-methylumbelliferone from 2′-(4-methylumbelliferyl)-α-D-N-acetylneuraminic acid (1 mM) by tetNA in 200 mM NaAc (pH 6.5), 2 mM CaCl2 and 1% butanol at 37 °C. Release of 4-methylumbelliferone was measured every 2 min for 1 h. One neuraminidase unit is defined as the amount of neuraminidase that releases 1 nmol of 4-methylumbelliferone per minute.

Virus like particles (VLP) were derived from the Hepatitis B core protein and have been described elsewhere^43^. Briefly, control VLP (previously referred to as VLP-1632) were derived from the gene encoding Hepatitis B core protein amino acids 1–163 followed by a cysteine residue. M2e-VLP (previously referred to as VLP-1965) are comparable to control VLP but contain three tandem copies of M2e fused N-terminally to Hepatitis B core (1–163), with the N-terminal M2e retaining the initiator methionine. Cysteine residues at position 17 and 19 in M2e are mutated to serine residues in the first two M2e copies to promote M2e-VLP formation upon rupture of *E. coli* cells. Control VLP and M2e-VLP were produced in and purified from BLR (Novagen) *E. coli*. Bacteria were transformed with either the control VLP or M2e-VLP and a single colony was picked, expanded overnight, and used to inoculate a 500-ml culture in Terrific Broth media (Invitrogen) and 50 μg/ml of ampicillin. Overnight cultures were harvested by centrifugation; cell paste was diluted to 10% solids in Tris-EDTA buffer (50 mM Tris-HCl, 10 mM EDTA, pH 8.0) and passed through a microfluidizer (Microfluidics, model 110Y) at 18,000 psi to disrupt cells. The lysate was clarified by centrifugation at 27,000× g for 60 min at 4°. Target protein was precipitated with 30% ammonium sulfate after continuous stirring for 60–90 min at 4 °C, collected by centrifugation at 10,000× g for 60 min at 4°, and resuspended in a minimal volume of Tris-EDTA buffer (50 mM Tris-HCl, 10 mM EDTA, pH 8.0). Suspension was dialyzed against the same buffer and clarified by centrifugation at 27,000× g for 30 min at 4°. Recombinant particles were purified from the cleared solution by gel filtration chromatography on a Sepharose CL-4B column, 2.6 × 95 cm (GE Healthcare Life Sciences), followed by flow-through purification on ceramic hydroxyapatite Type II resin with 20 μm particles size (BioRad Laboratories) in the presence of 20 mM sodium phosphate. Purified VLPs were dialyzed against 20 mM sodium phosphate, pH 6.8, followed by sterile filtration. The protein concentration was determined using a standard bicinchoninic acid assay (Pierce Thermo Fisher Scientific). Protein purity and integrity were visualized by NuPAGE 4–12% Bis-Tris 1.0 mm Gels in MES SDS Running buffer at 200 V constant voltage and stained with SimplyBlue SafeStain (all from Novex by Life Technologies). Endotoxin levels were tested by Endosafe –PTS cartridges (Charles River Laboratories).

Mice were vaccinated twice with 10 μg control VLP or M2e-VLP alone or in combination with one μg of triHA or tetNA as described in the text. Vaccine antigens were formulated in Sigma Adjuvant System (SAS, Sigma-Aldrich) according to the manufacturer’s instructions and administered subcutaneously. Mice were vaccinated twice with a three week interval and mock challenged or challenged with 0.1 LD_50_ of H1N1v three weeks after the boost injection. Seven weeks after the boost immunization mice were challenged with two LD_50_ of X47 virus. were three week intervals between primer and booster vaccinations, and between booster vaccination and homologous virus challenge (Figs 3 and 4).

### *N*-glycosylation analysis

N-glycosylation analysis was performed as described before^38^. Briefly, 2.5 μg tetNA, tetHA or a combination of both proteins was diluted to a concentration of 0.07 μg/μL in PBS (4 °C, 30 min) or in denaturing buffer (360 mM Tris-HCl pH 8.6, 8 M Urea, 3.2 mM EDTA, 55 °C, 30 min) before N-glycans were released using the on-membrane deglycosylation method and labeled with 8-aminopyrene-1,3,6-trisulphonic acid (Molecular Probes, Eugene, OR, USA)^38^. N-glycan samples and a reference dextran ladder (Sigma-Aldrich) were analyzed with a multicapillary electrophoresis-based ABI3130 sequencer.

### Serology

Blood was collected from the lateral tail vein and serum was prepared before vaccination, two weeks after primer and booster vaccination, and one week before challenge with X47 virus. Seroconversion was tested in ELISA. Hereto antigen-specific total IgG titers were determined using 96-well Maxisorp immune-plates (Nunc) coated over night with antigen: M2e peptide (SLLTEVETPIRNEWGCRCNGSSD, 2 μg/ml in carbonate buffer, 50 μl per well, 37 °C), triHA (1 μg/ml in PBS, 50 μl per well, 4 °C) or tetNA (1 μg/ml in PBS, 50 μl per well, 4 °C). After coating, plates were washed with wash buffer (PBS + 0, 1% Tween20) and blocked with 1% bovine serum albumin (Sigma-Aldrich) for 1 h at room temperature. For triHA and M2e ELISAs, 1/3 serial dilutions of mouse serum were incubated on coated plates for 1 h at room temperature, starting with 1/100 dilution. For tetNA ELISAs, a 1/100 dilution of each serum sample was used instead of a serial dilution series to check seroconversion. Sheep-derived anti-mouse serum conjugated with horseradish peroxidase (GE Healthcare) and tetramethylbenzidine substrate (Sigma-Aldrich) were used to detect antigen-reactive antibodies. Antibody titers against M2e and triHA are defined as the reciprocal of the highest dilution with an OD450 that is at least two times the OD450 of a pool of pre-immune serum samples.

Pooled serum samples were also evaluated for the presence of hemagglutination inhibition (HAI) activity, according to the WHO guidelines (WHO manual on animal influenza diagnosis and surveillance, 2002, Geneva; (WHO/CDS/CSR/NCS/2002.5) p28-39, http://whqlibdoc.who.int/hq/2002/WHO_CDS_CSR_NCS_2002.5.pdf). Briefly, sera were heat-inactivated at 56 °C for 30 min. Four volumes of receptor destroying enzyme (cholera filtrate from *Vibrio cholerae* culture fluid, Sigma-Aldrich) were added to each volume of mouse serum to remove aspecific agglutinins. To prevent aspecific binding to RBCs, sera were preabsorbed with 0.1 volumes of a 50% RBC suspension in PBS. Treated serum samples were mixed with 4 HAU of H1N1v in a final volume of 50 μl. After virus-antibody incubation for 1 h at room temperature, equal volumes of a 0.5% RBC suspension were added and HAI titers were recorded 30 min later. Endpoint titers were defined as the dilutions at which hemagglutination was inhibited completely.

RDE-treated serum pools were also tested in a microneutralization assay. Hereto two-fold serum dilutions were incubated with ten 50% tissue culture infective doses of H1N1v at 37 °C for 45 min. After incubation, monolayers of MDCK cells seeded in 96 well plates were washed with PBS and inoculated with the virus-serum mixtures. One hour post inoculation, serum-free growth medium supplemented with 2 μg/ml TPCK-treated trypsin was added and cells were further incubated at 37 °C for 72 h. Virus growth was determined by hemagglutination activity in the culture supernatants.

NA activity-inhibiting (NAI) antibody titers of pooled sera were quantitated by desialylation of immobilized fetuin as described^62^. Briefly, 96 well MaxiSorp plates (Nunc) were coated over night at 4 °C with 100 μl of fetuin in PBS (5 μg/ml). Serum samples were serially diluted in PBS enriched with 0.901 mM Ca++ and 0.493 mM Mg and incubated with 30 ng tetNA for 30 minutes at 37 °C. Serum-tetNA mixtures were then added to the fetuin-coated 96-well plates and incubated for 1 h at 37 °C. After washing, desialylated fetuin was detected by adding horseradish peroxidase-labeled peanut agglutinin (2.5 μg/ml, Sigma) for 1 h at room temperature. After washing, tetramethylbenzidine substrate (Sigma-Aldrich) was added. After five minutes, the reaction was stopped by adding 1 M sulfuric acid and optical densities were measured at 450 nm with a reference at 650 nm. NAI titers are defined as the reciprocal of the highest dilution with an OD450 that is at least half the OD450 of a pool of pre-immune serum samples.

### Lung virus titers

Three mice of each group were euthanized at the indicated day post infection (dpi). Lungs were removed aseptically and 10% (w/v) lung homogenates were made in PBS. Lung homogenates were cleared by centrifugation (400 g, 10 min, 4 °C) and stored at −80 °C. Infectious virus titers were determined on MDCK cells in serum-free TPCK-treated trypsin-containing medium. Chicken RBC agglutination activity in the cell supernatant at 7 dpi was used to calculate endpoint virus titer according to the method of Reed and Muench^63^. Lung virus loads are expressed as tissue culture infectious dose 50 (TCID_50_) per gram of lung extract.

### Cytokine/chemokine measurements

Twenty five μl of undiluted cleared lung homogenates prepared for lung virus titration were used for measurement of chemokines and cytokines using Luminex^®^-based Milliplex MAP Mouse Cytokine/Chemokine multiplex assay (EMD Millipore) according to the instructions of the manufacturer using a Bio-Plex^®^ 200 system (Bio-Rad).

## T cell analysis

### Intracellular staining of IFNγ

Spleens of six mice/group were removed aseptically at 10 dpi and splenocytes were prepared. RBCs were lysed with NH_4_Cl solution and 2×10^6^ splenocytes were restimulated for 6h with MHCI-binding H2d-restricted NP-derived TYQRTRALV peptide (5 μg/ml, NP155) in 100 μl culture medium in the presence of brefeldin A (Golgiplug, BD) for intracellular staining of IFNγ. T cells were immunophenotyped after blocking of Fc receptors with anti-CD16/CD32 (Fc Block, BD) by anti-CD3ε-APC-Cy7 (BD) and anti-CD8α-FITC (BD). After staining of cell surface markers, cells were fixed and permeabilized with the Cytofix/Cytoperm kit (BD) according to the manufacturer’s protocol. Intracellular IFNγ was stained with anti-IFNγ-AF647 (BD). NP-specific T cells were identified by adding PE-conjugated pentamers associated with the NP155-peptide (15 μl of 0.05 mg/ml solution/10^6 ^cells, ProImmune) to the culture medium during restimulation. Background signals were reduced by creation of a dump channel for dead cells (Live/Dead Fixable Aqua dead cell stain kit, Invitrogen) and for MHCII-eFluor450 (eBiosciences) or CD19-PERCP-Cy5.5 (BD). Cells were analyzed using a LSRII flow cytometer (BD) with FACSDiva software (BD). Analysis of flow cytometric data was with FACSDiva (BD) or FlowJo (Treestar) software.

### *In vivo* killing assay

*In vivo* clearance of NP155-peptide pulsed target cells was used to estimate killing capacity of NP-specific T cells. Briefly, splenocytes of naïve BALB/c mice were pulsed for 1h at 37 °C with NP155-peptide or with an irrelevant peptide F85 (KYKNAVTEL), derived from the F protein of respiratory syncytial virus. Peptides were added to the culture medium (RPMI1640 + 10% fetal calf serum) at a concentration of 10 μg/ml. After washing with PBS, NP155-pulsed cells or F85-pulsed cells were stained with either 10 μM or 1 μM carboxyfluorescein diacetate succinimidyl ester (CFSE, Invitrogen) for 15 min at 37 °C in the dark. After washing and resuspending in PBS, NP155- and F85-pulsed cells were mixed at a 1:1 ratio and 10^7 ^cells were injected intravenously into receiver mice. After the indicated time (6 h or 19 h), splenocytes were prepared from receiver mice and analysed on a LSRII flow cytometer (BD). The ratio of the number of NP155-pulsed splenocytes over the number of F85-pulsed splenocytes was calculated and used as a measure for the *in vivo* NP155-specific killing capacity.

### NP-specific T cells in circulation

NP155-peptide specific T cells were quantitated in pooled blood samples (n = 3/pool) collected from the lateral tail vein at the indicated time points. Hereto 100 μl blood was collected in 30 μl 100 mM EDTA. RBCs were lysed in 500 μl RBC NH_4_Cl buffer and resulting cells were washed in PBS. Fc receptors were blocked (Fc Block, BD) and live NP155-specific CD8+ T cells were quantified by staining with Live/Dead (Fixable Aqua dead cell stain kit, Invitrogen), surface markers (CD3ε-APC, CD8a-PERCP, B220-AF700) and NP155-peptide associated PE-conjugated pentamers (ProImmune) in PBS + 1% BSA followed by analysis on a FACSverse flow cytometer (BD). A dump channel was made with B220-AF700 and Live/Dead stain to reduce back ground staining.

### Inducible bronchus-associated lymphoid tissue analysis

Lungs were isolated, homogenized in PBS and forced through a 40 μm cell strainer to obtain single cell suspensions. After blocking of Fc receptors (Fc Block, BD), cells were stained for viability (Live/Dead Fixable Aqua dead cell stain kit, Invitrogen) and surface markers (IgM PERCP-Cy5.5, IgD-PE, Fas-PE-Cy7, B220-AF700 and GL7-FITC). The number of live germinal center B cells (IgM-IgD-Fas + GL7+) was used to estimate presence of iBALT in lungs using a BD LSRII flow cytometer. For histology, lungs were inflated with a 1:1 mixture of PBS and Neg-50 cryo-medium (Prosan). Lungs were snap-frozen in liquid nitrogen after excision and stored at −80 °C. Cryo-sections of 5 μm were stained with hematoxylin/eosin.

### Statistics

Statistical analyses were performed with Graphpad Prism version 6.00 for Windows (GraphPad Software, San Diego California; www.graphpad.com) and with the R language and environment for statistical computing, R Development Core Team, 2009 (R Foundation for Statistical Computing, Vienna, Austria (ISBN 3-900051-07-0, URL http://www.R-project.org). The statistical tests used for computing significance levels as well as the significance levels are mentioned in the figure legends.

## Additional Information

**How to cite this article**: Schotsaert, M. *et al.* Long-Lasting Cross-Protection Against Influenza A by Neuraminidase and M2e-based immunization strategies. *Sci. Rep.* **6**, 24402; doi: 10.1038/srep24402 (2016).

## Supplementary Material

Supplementary Information
