# Supplementary material for: Long-Lasting Cross-Protection Against Influenza A by Neuraminidase and M2e-based immunization strategies
Source: Sci Rep. 2016 Apr 13;6:24402. doi: 10.1038/srep24402 (PMC4829898; doi:10.1038/srep24402)
Supplement: Supplementary Information [file srep24402-s1.doc]

**Long-Lasting Cross-Protection Against Influenza A by Neuraminidase and M2e-based immunization strategies**

Michael Schotsaert,^a,b,*^ Tine Ysenbaert,^a,b^ Anouk Smet,^a,b^ Bert Schepens,^a,b^ Dieter Vanderschaeghe,^a,c^ Svetlana Stegalkina,^d^ Thorsten U. Vogel,^d^ Nico Callewaert,^a,c^ Walter Fiers,^a,b^  Xavier Saelens,^a,b^

**Supplementary information**

**Supplementary Figure 1** Concentrations of the indicated cytokines and chemokines measured in cleared lung homogenates from individual mice (n=3/group) harvested on day 6 after mock infection or infection with 0.1LD_50_ of H1N1v for experiment 1. The vaccination and H1N1v challenge (mock: “-“; H1N1v infection: “+”) status of the mice is indicated below each column of graphs. Cytokine and chemokine expression levels were determined using a 19-plex cytokine bead array and are depicted as average concentrations in pg/ml of individual mouse samples. Error bars represent standard deviations.

**Supplementary Figure 2** Concentrations of the indicated cytokines and chemokines measured in cleared lung homogenates from individual mice (n=3/group) harvested on day 6 after mock infection or infection with 0.1LD_50_ of H1N1v for experiment 1. The vaccination and H1N1v challenge (mock: “-“; H1N1v infection: “+”) status of the mice is indicated below each column of graphs. Cytokine and chemokine expression levels were determined using a 19-plex cytokine bead array and are depicted as average concentrations in pg/ml of individual mouse samples. Error bars represent standard deviations.
